# A governance perspective on agri-environmental schemes: Actors, roles, and barriers

**DOI:** 10.1007/s13280-025-02182-0

**Published:** 2025-04-19

**Authors:** Diana Borniotto, Clémentine Antier, Philippe V. Baret

**Affiliations:** Sytra, Earth and Life Institute, UCLouvain, Croix du Sud 2, L7.05.14, 1348 Louvain-la-Neuve, Belgium

**Keywords:** Agri-environmental schemes, Barrier analysis, Common agricultural policy, Environmental performance, Multilevel governance framework

## Abstract

Agri-environmental schemes (AES) carry significant environmental expectations. However, their environmental performance has often been questioned. Existing research has tackled the issue from ecological and economic perspectives. This study aims to provide a complementary governance perspective. It first develops a theoretical framework to analyze AES governance through a multilevel lens, identifying three distinct levels based on different actors’ roles: macro (setting policy boundaries), meso (implementation of the policy framework), and micro (farmer actions). Second, this study develops an analytical framework to investigate barriers hindering AES environmental performance and their linkage to AES governance. The combined frameworks have been tested in a regional case study in the Hauts-de-France region. A list of 40 barriers is identified, spanning organizational, power dynamics, and financial issues. Barriers are not confined to farm or policy scale, distributing unevenly across governance levels, with different agencies across roles and actors in unlocking the AES performance.

**Supplementary Information:**

The online version contains supplementary material available at 10.1007/s13280-025-02182-0.

## Introduction

The environmental performance of Agri-Environmental Schemes (AES), defined as the extent to which the environmental aim of an AES can be achieved (Schomers and Matzdorf 2013), has often been questioned. Such limitation has been grounded in a systematic lack of achievement of their long-term environmental objectives, such as biodiversity protection, habitat preservation, and climate change mitigation (Primdahl et al. 2003; Kleijn et al. 2006; Matzdorf and Lorenz 2010; Pe’er et al. 2014; Galler et al. 2015; Leventon et al. 2017; Guyomard et al. 2023). Under the EU Common Agricultural Policy (CAP), AES serve as a key policy instrument for biodiversity conservation and climate protection (European Environment Agency 2019). These schemes entail a series of measures, each designed to target specific farming practices or landscape features. Examples include reducing environmental risks (e.g., reducing fertilizers or pesticides), protecting nature (e.g., leaving winter stubbles in intensive arable areas to provide food for birds, flower strips for pollinators), and preserving traditional farming practices (e.g., extensive grazing, traditional vineyard management, agropastoralism) (Bazzan et al. 2022). AES are implemented across all EU Member States (MS) under different contractual arrangements (e.g., action-based, result-based, collective, or value chain contracts) that shape the payment mechanism of AES (Bazzan et al. 2022; Sattler et al. 2023).

The scientific literature has extensively debated the underlying factors of AES environmental shortfalls such as the lack of clear and quantifiable AES objectives (Kleijn et al. 2006), spatial mismatches (Toderi et al. 2017; Früh-Müller et al. 2019; Rotchés-Ribalta et al. 2021), the simplistic conceptualization of farmer behavior in the design AES (Hasler et al. 2019; Brown et al. 2021), high transaction costs (Falconer 2000; Weber 2015), opportunity costs, and low payment levels (Bartkowski et al. 2023). Those critiques have been formulated under either ecological or economic perspectives—mainly focusing on environmental impact assessment or cost–benefit analysis—as pointed out by Uthes and Matzdorf (2013) and more recently by Bazzan et al. (2023a,b). Some scholars have focused on aspects related to their governance, exploring the interconnection between policy and farm scales (hereinafter referred to as AES governance literature) (Lowe et al. 2002; Beckmann et al. 2009; Matzdorf et al. 2013; Mettepenningen et al. 2013; Meyer et al. 2015, 2018; Bazzan et al. 2023a,b; Sattler et al. 2023). The AES governance literature, even if only recently targeted as a research topic, presents an extensive body of research focusing on linking AES governance and AES environmental outcomes (Fairbrass and Jordan 2001; Smits et al. 2008; Meyer et al. 2015; Bazzan et al. 2023a,b). Their findings confirm previous evidence on AES environmental shortfalls, yet based on different underlying factors, such as stakeholder involvement (Beckmann et al. 2009; Leventon et al. 2017; Sattler et al. 2023), knowledge exchange (Bazzan et al. 2023a,b), flexibility in implementation, institutional design, institutional arrangements, and specific design rules employed (Mettepenningen et al. 2013; Schomers et al. 2015; Toderi et al. 2017; Westerink et al. 2017; Lien et al. 2018; de Vries et al. 2019; Bazzan et al. 2022), power dynamics across stakeholders (Beckmann et al. 2009; Benoit and Patsias 2017). Other studies have emphasized the role of decentralization of governance and subsidiarity of decision-making (Lowe et al. 2002; Beckmann et al. 2009). However, such literature struggles to provide a systematic analytical framework to describe how AES environmental shortfalls and the related underlying factors affecting AES environmental performance are linked to the governance framework.

Against this background, the study addresses the following research question: Which factors linked to AES governance hamper the environmental performance of AES?

To address this question, we develop a theoretical and analytical framework to examine AES governance structures and identify the factors that hinder their environmental performance, hereinafter referred to as barriers. The paper is structured as follows: “Theoretical background” section provides the theoretical foundation, identifies the research gap, and introduces the theoretical framework and the research design steps, along with the case study selected for empirical analysis. “Materials and methods” section outlines the analytical framework, which consists of two components, one focusing on the governance structure of AES and the other on the barriers that influence the link between governance and environmental performance. “Results” section presents the key findings related to governance structures and barriers. “Discussion” section discusses the implications for AES governance within the case study and in the broader context of EU regions. Finally, “Conclusions” offers the main conclusions of the study.

## Theoretical background

### A research gap in AES governance literature

Defining governance is often complex and does not stop to one meaning and usage (Hill et al. 2016). The AES governance literature is no exception and, even if not providing a definition, converges on several common elements defining the concept of governance: a clear goal or priority to be achieved, a plurality of actors from both public and private entities, dynamic interactions among these actors (Beckmann et al. 2009; Westerink et al. 2017; Bazzan et al. 2023a,b; Sattler et al. 2023), and the presence of institutions, rules, and institutional arrangements that shape these interactions (Meyer et al. 2015; Lien et al. 2018; Bazzan et al. 2023a,b). Consistent with the multilevel governance framework prevalent in EU public policy studies (Hooghe and Marks 2001; Jordan 2001; Hooghe and Marks 2003; Bache et al. 2016), AES governance research also underlines that decision-making occurs at various levels, affecting both the design features (e.g., spatial targeting, payment structure, duration of contracts) (Bazzan et al. 2023a,b) and the overall governance structure (Beckmann 2009).

Multilevel governance means interactions between actors encompassing cooperation, negotiation, and potential conflict and institutional arrangements, including rules, procedures, and funding mechanisms, take place across levels and are key in shaping AES policy outcomes (Mettepenningen et al. 2013). Some studies employ the term “multilevel governance,” for example, McCarthy et al. (2018), directly, whereas others explore multilevel dynamics indirectly Beckmann et al. (2009).

The AES governance literature employs geographical and actor-based definitions of “levels” to outline the AES governance. However, these definitions lack standardization, as the specific administrative levels vary across sources and geographical contexts and the categorization of actors (public, private, civil society) can be fluid, with hybrid forms existing. This lack of standardization mainly relates to the EU subsidiarity principle governing the AES design, under which the governance structures overseeing the schemes vary significantly across MS. The subsidiarity principle, introduced in the early 90 s during a change in the EU political *zeitgeist* and targeting political areas such as the environment (Jordan and Jeppesen 2000), states that decision-making should take place at the lowest administrative level consistent with effective actions (Van Kersbergen and Verbeek 1994). This shapes the governance under which the CAP policy framework is implemented within each MS, and consequently AES. Some MS have opted for a centralized governance model, while others have embraced a regionalized approach, defining the general approach to AES governance (Beckmann et al. 2009). Additionally, different AES contract types could define the mandates given to various actors, adding another layer of potential heterogeneity across contexts. Therefore, defining levels based on geographical or actor-based criteria would not ensure the external validity of such a framework across the diverse national contexts within the EU, failing to provide a common framework for the heterogeneous nature of AES implementation processes across EU regions and MS.

### The proposed theoretical framework: AES multilevel framework

The AES governance within the European Union, while resulting in diverse implementation across MS, still requires adherence to a common set of steps. This is due to three main factors: (i) the shared objectives of AES (e.g., biodiversity protection) as a result of EU regulation (i.e., Regulation (EU) 2021/2115), (ii) the fact that AES are part of public policy, which brings common institutional setting and institutional arrangements, and (iii) AES across all MS—as voluntary-based subsidies—share a common goal of influencing farmers’ behavior at the farm level, striving for broader impacts on ecosystems. Due to these commonalities, each MS is constrained by a predefined set of steps linking the public policy framework to the farm-level dynamics. From an institutional perspective, we argue that the decision-making processes defining the AES governance can be divided into three levels: (i) a strategic level of AES, where the “rules of the game” are established, setting therefore the boundaries and defining the characteristics of the policy framework, (ii) an implementation level, focusing on translating the broad policy objectives into tangible actions on the ground ensuring its implementation at both national and regional scales, and (iii) an operational level linked to the receiving end of AES policies—primarily farmers and land managers—whose decisions and actions ultimately affect the success or failure of these schemes at farm scale.

Table 1 gives an overview of AES institutional multilevel governance framework.

**Table 1:** The AES institutional multilevel governance framework

Governance level	Description
*Macro-level: setting the boundaries*	Delineates the overarching boundaries set by regulations governing AES policies framework
*Meso-level: ensuring implementation*	Ensures the implementation of AES coherent with the overarching public policy frameworks defined at macrolevel. This level interfaces with either the macrolevel or the microlevel under distinct mechanisms
*Micro-level: adopting parties*	Focuses on the direct recipients of the policy framework. It refers to the AES adoption and related changes in farming practices and landscape features

The theoretical framework is designed to define the *structure* of AES governance, providing the necessary foundation to unravel its associated *process,* which is here defined as the complementary interactions among various actors within a set of institutional arrangements aimed at a common goal. The AES governance process requires operationalizing the theoretical framework to identify the interactions linking the levels both horizontally (i.e., within levels) and vertically (i.e., across levels).

### The research design steps

The theoretical framework serves as a basis for designing the research. Two steps are defined as (i) the development of a multilevel governance framework for AES governance; and (ii) the identification of barriers in the governance process.

The first research design step operationalizes the multilevel governance framework in the AES context; we define roles, assign them to mandated actors, and cluster them by governance level, aligning with common objectives.

It draws on the concept of roles as proposed by Sattler et al. (2023). In their research on innovative AES contracts, the authors utilize the concept of “roles” to analyze the distribution of responsibilities among the actors involved in AES governance. They define roles as “a set of recognizable activities used by an actor to address recurring situations,” (Sattler et al. 2023) building on the work of Westerink et al. (2017). Within the AES framework, these roles clarify how various actors—such as farmers, government agencies, NGOs, and private companies—contribute to AES governance. The roles encompass activities such as contract design, implementation coordination, funding provision, outcome monitoring, and policy advocacy. Their application of roles is rooted in the broader context of institutional analysis, particularly the Institutional Analysis and Development (IAD) framework developed by Elinor Ostrom (Ostrom 2009).

For the second research design step, the study mobilizes the concept of barriers to describe the underlying factors affecting AES environmental performance. Scholars have approached a wide array of topics through the lens of barriers, including pesticide usage (Cowan and Gunby 1996; Hofmann et al. 2023), cooperative dynamics (De Herde et al. 2022), technological innovations and genetic engineering (Vanloqueren and Baret 2009), crop diversification (Meynard et al. 2018; Morel et al. 2020; Weituschat et al. 2022), and value chain dynamics (Williams et al. 2024). This study builds upon the methodological insights derived from this literature and provides an analytical framework to link barriers to AES governance. To achieve this, the study applies a multilevel framework to AES governance, identifying where barriers arise in the governance process and which actors, across different levels, have agency over them.

### The case study

This study adopts a case study approach focused on the AES governance in the Hauts-de-France region. The case study was selected for three reasons. Firstly, a regional approach enables engagement with a diverse set of actors operating at this scale, most of whom are included in the mesolevel—i.e., the level of implementation of the AES policy framework. These actors often have an in-depth understanding of the institutional mechanisms characterizing the AES governance, and they interact with both macro and microlevels, thereby providing a comprehensive view of the AES governance process.

Secondly, the Hauts-de-France is a predominantly agricultural region with high production levels and significant environmental challenges. Despite efforts to support a sustainable environmental transition through regional and national initiatives, the agri-food production system generates substantial negative environmental impacts, greatly affecting the local social-ecological systems (Martin et al. 2021). In 2022, the AES subsidies allocated to the region amount to roughly 14 million euros, against 255 million euros for direct payments and 31 million euros coupled income support (for livestock) (Agreste 2024).

Thirdly, from a national perspective, France presents an interesting governance structure. Historically, the French governance is largely centralized but recent efforts, particularly during François Hollande’s presidency (from May 2012 to May 2017), support a process of decentralization. This combination of centralized governance tradition and emerging decentralization makes Hauts-de-France an interesting case for studying the AES governance, which is inherently driven by the principle of subsidiarity.

## Materials and methods

### The analytical framework

**Fig. 1:**
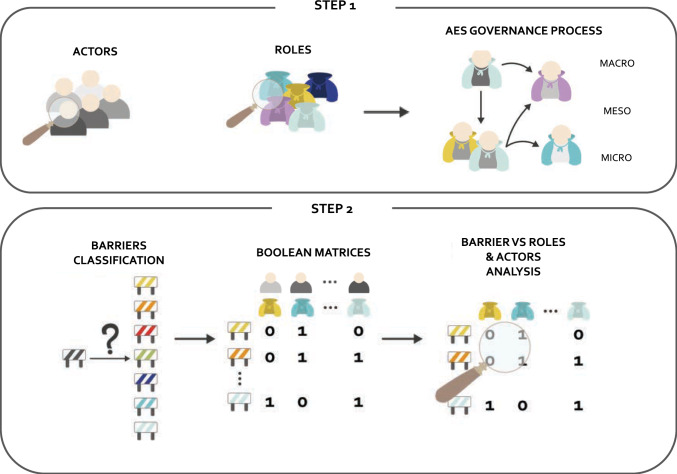
Analytical framework composed by two complementary steps, one targeting the AES governance and the other the barrier analysis

The analytical framework includes two successive steps, summarized in Fig. 1. The first step aims to highlight the key components of AES governance by identifying actors and their roles and eventually delineating the AES governance process linking them. The second step involves identifying and characterizing barriers hindering AES environmental performance and their linkage to AES governance. For this purpose, barriers are classified into categories and linked to roles and actors. Those links are eventually analyzed.

#### Step 1: The AES governance process

This step strongly relies on the methodology developed by Sattler et al. (2023). For this, the AES governance is defined through the same dimensions: types of actors and their roles. The authors’ categorization provides a comprehensive list of roles within AES contract governance that was adapted for this study (see Table S2.1 in Appendix S2). The authors developed a list of roles for innovative AES contracts (i.e., result-based, collective, land tenure, and value chain AES contracts) in the EU, encompassing a wide range of actors from the private, public, and third sectors, involved in the AES institutional governance process. We adapted the scope of this list as this research focuses exclusively on action-based mainstream AES (Sattler et al. 2023). An iterative process across the research team and participants of both interviews and focus group, which will be outlined in “Data collection and analysis” section, allowed for the identification and description of (i) each role, (ii) the actors performing them, and (iii) the nature of the interactions occurring across these actors and roles. These three elements build the AES governance process described in the result “The AES governance structure and process” section.

#### Step 2: The barrier analysis

This step develops a barrier analysis to target the link between underlying factors hindering AES environmental performance and the AES governance. It entails the development of two Boolean matrices mapping the links between barriers and AES governance roles and actors.

During this phase, we compiled the list of barriers collected during the interview phase, described in the “Data collection and analysis” section; barriers were grouped into categories (i.e., technical, power dynamics, organizational, knowledge, institutional, financial, cultural). To link the list to the AES governance, two Boolean matrices are built linking each barrier to (i) the actor(s) it involves and (ii) the role(s) it touches. Barriers are linked to roles and actors answering two distinct questions: (i) across which role(s) does the barrier unfold (ii) which actor(s) has direct agency over the barrier.

### Data collection and analysis

#### Data collection

Primary data were collected from four complementary sources: desktop research, expert consultations, semi-directive interviews, a seminar, and a focus group.

Desktop research was conducted as a first step to collect background information on the AES implementation process at the EU and MS levels and on the case study regional context. We consulted: five scientific publications, three policy documents, six policy reports, and one methodological guideline (full list provided in Table S1.1 in Appendix S1). This preliminary step was also used to identify and select experts for the expert consultation.

The expert consultations were conducted in March 2023 with three experts (i.e., individuals with extensive knowledge and understanding of regional sociopolitical and environmental dynamics) who were asked to provide (i) a brief description of the regional context, along with reports and literature references, and (ii) relevant contacts to be used for the interviews. The experts were selected based on their knowledge of regional networks, their experience in the research area, and their availability.

This allowed us to complete the desktop research and yield the first list of key actors for interviews. Actors were selected based on their active (or past) role in the AES governance in the region. Snowball sampling technique (Naderifar et al. 2017) was adopted to complete this list. A total of 17 participants were interviewed (overview in Table S1.2 in Appendix S1).

Throughout the interview process (March 2023 to August 2023), participants were asked to: (i) introduce themselves, their organizational affiliation, and geographical competence (e.g., national, regional); (ii) outline the role of their respective organizations; (iii) list and describe the nature of interactions with other relevant actors, and (iv) provide their understanding on the barriers hindering AES environmental performance (full interview protocol is provided in Table S1.3 in Appendix S1).

Data collection allowed the drafting of two outputs:

1. AES governance insights: providing information on the actor’s types, their roles, and the interconnections across actors
2. List of barriers: providing information on the barriers hindering AES environmental performance

Data validation for the AES governance process, including actors, roles, and interactions, was conducted during an online seminar where all participants of the interview phase were invited and could provide feedback.

Data validation for the list of barriers was ensured through a two-step approach. The first interviews were analyzed chronologically, and the lists were expanded until no further data acquisition on the specified parameters emerged, according to the principle of data saturation (Saunders et al. 2018). Second, the two lists were validated through a focus group where all participants were asked to review the completeness of both lists. Some of the participants could not join, and for those an email was sent to collect their feedback.

#### Data analysis

**Fig. 2:**
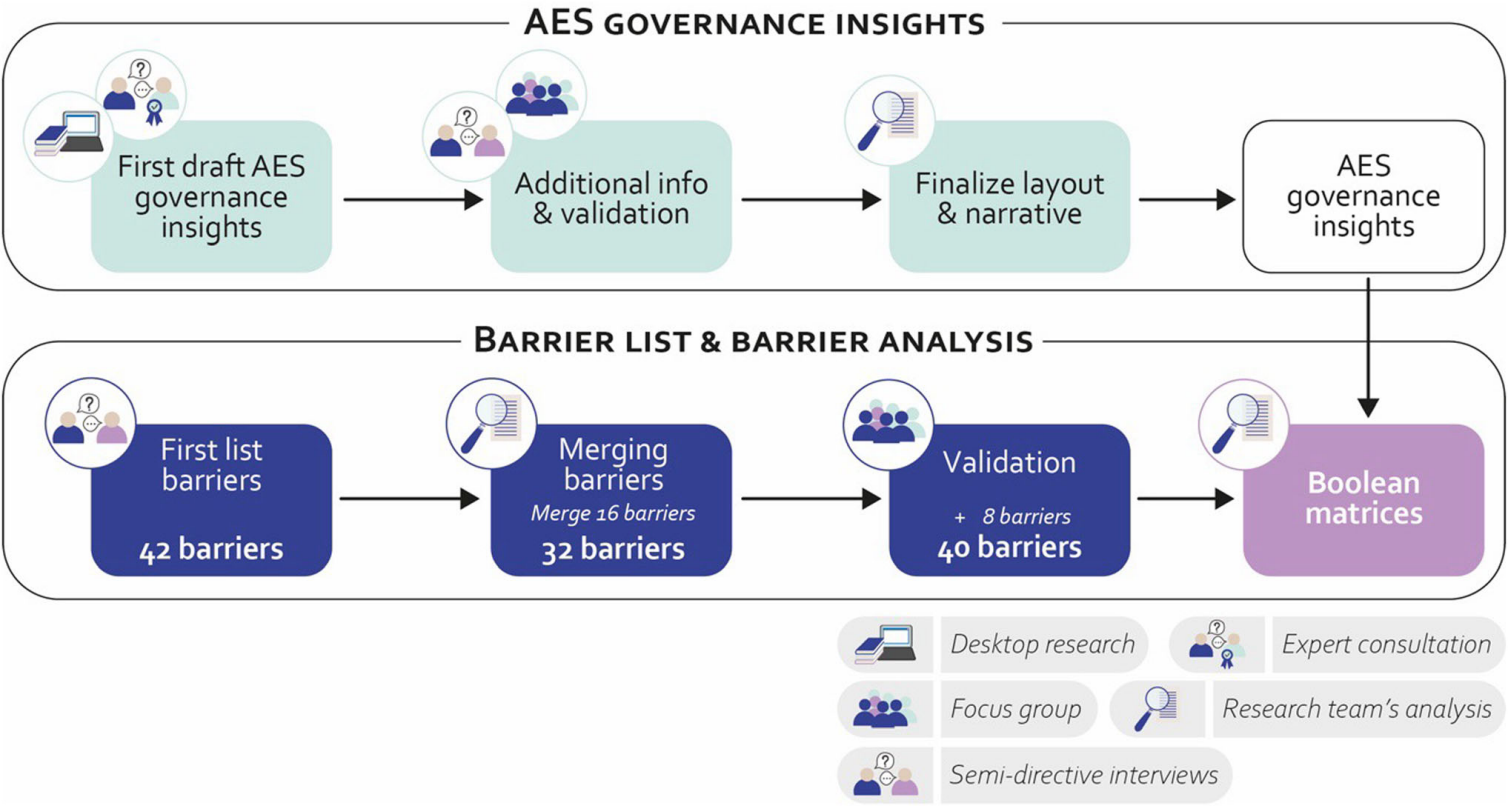
Data collection design and data analysis conducted in the regional case study

Data analysis was conducted differently for the two methodological steps, which are summarized in Fig. 2.

**Fig. 3:**
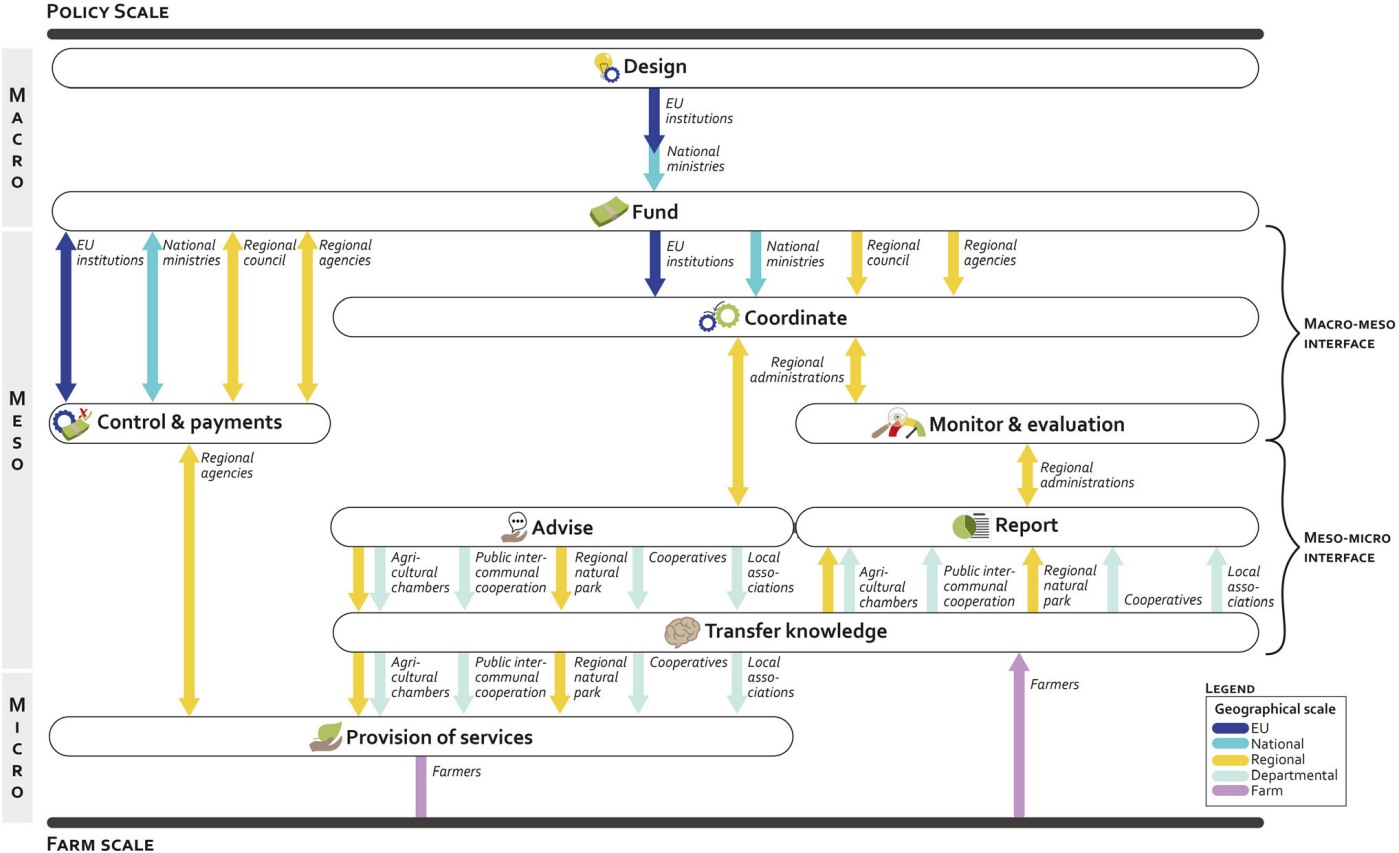
AES roles, actors, and multilevel governance framework in Hauts-de-France region. Roles are represented by circled boxes, arrows represent actor types, and their associated colors the geographical scale they operate in

Step 1 aimed to develop a descriptive representation of the AES governance process. Initially, a preliminary version of the AES governance insights was drafted based on information retrieved through the desktop research. Subsequently, insights retrieved during the interviews provided complementary information to refine the draft. This revised draft was then discussed and validated during a seminar. The research team developed a table summarizing the main information retrieved in the draft (Table S2.2 in Appendix S2) that was used, along with the narrative built throughout the interviews and the seminar, to finalize the layout of the final version, presented Fig. 3 in the result section.

Step 2 required a more elaborate analytical approach. Barriers collected through each interview have been listed, allowing us to retrieve 42 unique barriers. Following this, a merging phase consolidated barriers addressing similar issues, reducing the list to 32 barriers and assigning new names to the merged barriers. The focus group conducted with a subsample of the interview’s participants allowed us to validate the 32 barriers and add 8 more barriers. We opted for conventional qualitative content analysis (Hsieh and Shannon 2005) to group the barriers under specific categories. The research team organized the 40 identified barriers into seven categories based on thematic similarity, ensuring that the clustering reflected underlying patterns across the barriers.

Eventually, the research team built two Boolean matrices (a matrix consisting only of 0 s and 1 s, representing binary values) to link each barrier to roles and actors. The value “1” was assigned where the link occurred, and the value “0” otherwise. Two researchers conducted the analysis independently, following a common guideline. Later, three working sessions at the research team level were organized to discuss the differences and finalize the matrices. All data analysis steps can be found in Appendix S3.

## Results

### The AES governance structure and process

#### The roles and actors behind the AES governance

Nine roles and 12 actor types are identified as participating in the AES governance in the Hauts-the-France region (Table 2). The roles range from designing and funding the AES, coordinating related activities, organizing control and payment, monitoring and assessing progress, advising on farming practices, reporting on actual implementation, and providing services for which the AES payments were originally designed. A description of each actor type is provided in Table S2.3 in Appendix S2.

**Table 2:** The roles and actor types of the AES governance in the case study Hauts-de-France

Roles in the AES governance	Actor types in the AES governance
1. Design	1. EU institutions
2. Fund	2. National ministries
3. Coordinate	3. Regional administrations
4. Control & payment	4. Regional council
5. Monitoring & evaluation	5. Regional agencies
6. Advise	6. Control & payment agencies
7. Report	7. Agricultural chambers
8. Transfer knowledge	8. Intermunicipal cooperation
9. Provision of services	9. Regional natural park
	10. Cooperatives
	11. Local associations
	12. Farmers

#### The AES governance process linking actors and roles

This section presents the AES governance process in the regional case study. Figure 3 showcases the interconnection of roles, actors, and geographical scale at stake between the policy and the farm scales. The roles and the implication of actors within each role are explained below.

Under the AES public policy framework, the overarching legislative framework is designed firstly by EU institutions that set requirements and constraints to be implemented across all MS, as procured in Article 70 (EU Regulation, 2021/2115). Under the green architecture, the regulation establishes that AES payments should commit to farming measures that go beyond what is included in the Good Agricultural and Environmental Conditions (GAEC) and the Statutory Management Requirements (SMR). It also mentions that MS may promote and support collective schemes and result-based payments schemes to encourage farmers or other beneficiaries to deliver a significant enhancement of the quality of the environment at a larger scale.

The EU principle of subsidiarity—defined by Article 5 of the Consolidated Version of the Treaty on European Union (2016)—plays a significant role in the AES design, granting MS substantial autonomy in defining various aspects of AES, including their objectives, concrete targets, and expected outcomes. Such principle aims to enable tailored solutions to regional agricultural challenges, leaving decisions to MS, regional, or smaller subunits (Van Kersbergen and Verbeek 1994). In France, the Agricultural & Food Ministry is appointed the mandate to draft a proposal for AES, along with all other policy instruments of both pillar I and II of the CAP (e.g., direct payments, LEADER programs). The proposal is then discussed in stakeholders’ dialogues, primarily involving unions and farmers’ organizations, and with other Ministries, such as the Environment and Ecology Ministries. Once these negotiations are concluded, and with the approval of the Prime Minister, the proposal is finalized, and the policy document is then sent to the EU Commission, for EU-MS negotiations. The final draft of the policy document on AES (along with all other CAP policy instruments) is highly detailed and provides the list of all AES measures authorized in the national territories, along with payments per ha or per farm (depending on the type of measure). For the programming period 2023–2027, the AES design is included in the CAP Strategic Plans (Ministry of Agriculture and Food Sovereignty 2022).

The AES *funding* in France is structured across multiple tiers. It includes EU contributions channeled through the European Agricultural Fund for Rural Development (EAFRD). Additionally, as mandated by EU laws, the Ministry of Agriculture and Food provides complementary funding for AES implementation. Furthermore, regional entities like water agencies, which operate in all French regions, allocate portions of their financial resources to support the AES program, ensuring the achievement of some of their objectives.

Having established AES design and funding, MSs can appoint diverse actors at different geographical scales with the mandate to *coordinate* AES activities. In France, the AES are managed at regional level by the regional administrations through the Agri-Environmental and Climatic Projects (AECP). The Agricultural Ministry delegates the regional administration to draft public calls for AECPs across the region, for which local partners (e.g., agricultural chambers, natural parks, local NGOs, cooperatives) can apply as project leaders. If selected, the local partner provides a detailed plan of the AES measures to be implemented in the area through the AECP. The AES measures are chosen from a predefined list included in the CAP National Strategic Plan for the 2023–2027 programming period. The selected AES measures should align with local environmental threats previously identified at local and regional levels (Ministry of Agriculture and Food Sovereignty 2022).

Once granted the AECP mandate, local partners must develop a farming *advising* campaign strategy, ensuring a *knowledge transfer* to achieve expected changes at farm level. Local partners are also required to *report* frequently on the activities conducted under their AECP, ensuring a *monitoring & evaluation* framework, for which the regional administrations are responsible for.

Once subsidies become available under the AES measures within AECP, farmers become eligible to apply for funding. AES contracts span five years, providing annual payments for the implementation of specific farming practices (e.g., delayed mowing season) or the introduction of landscape features (e.g., hedges, flower strips) within designated areas of the farm.

Before applying for funding, farmers are required to compile a diagnostic to ensure that the AES measures planned on the farm align with the overarching requirements and priorities outlined in the related AECP. *Control* agencies conduct an initial screening to assess the completeness and compliance of the application form and the provided diagnostic. After compliance is approved, *payment* agencies oversee the processing of the farmer’s application. Additional controls may be conducted to verify ongoing compliance throughout the five-year period. Farmers are required therefore to keep informed competent authorities (i.e., *knowledge transfer role*) on the various indicators to monitor the subsidies’ use.

National authorities define payment rates for each AES measure. They are aligned across all regions and based on the foregone incomes associated with implementing AES measures EU Regulation (2021/2115).

#### The AES governance process through the multilevel framework

**Fig. 4:**
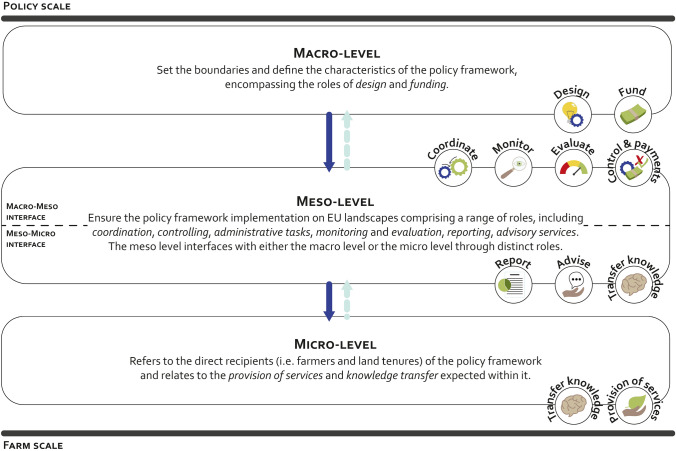
AES roles defining each level of the AES multilevel governance framework

The multilevel governance framework building upon three main levels (i.e., macro-, meso-, and microlevel) now can be mobilized to organize the roles that transform a policy framework in actions at farm scale through the AES governance process (Fig. 4). The macrolevel, which sets the boundaries of the policy framework, accounts for both the design and funding roles, as they are the main responsible for shaping the policy framework. The mesolevel ensures the implementation of the AES policy framework. It comprises a vast array of roles, such as the coordination by regional public administrations, the monitoring and evaluation by control agencies, and the advising activities by various actors such as agricultural chambers. This level is characterized by two main interfaces, one with the macrolevel, grouping roles such as coordination, monitoring, and evaluation that interact with roles at the macrolevel, and one with the microlevel, ensuring both advising activities and knowledge transfers. The microlevel focuses on the direct recipients of the policy framework and relates to the provision of services and knowledge transfer expected within the public policy framework. It refers to the AES adoption and related changes in farming practices and landscape features.

### The barrier analysis

#### Overview of the barriers

We identified 40 barriers hindering AES environmental performance. These barriers include issues such as the administrative burden associated with AES design, limited budget allocation, insufficient coordination among entities managing AES funding, and the underappreciation of AES-related food production within value chains.

We identified seven categories under which we grouped the 40 barriers (Table 3).

**Table 3:** Categories of barriers, brief description, and listing of specific barriers per category

Category of barriers	Description	Specific barriers
Technical	Barriers referring to technical aspects impeding the AES smooth and effective implementation. This category relates to both administrative and farming aspects of AES	1. Complex and time-consuming administrative tasks for subsidies-related documents for both farmers and public administrations
		2. Nonrealistic AES set of requirements to be implemented at farm level
		3. High specialization of value chain and high concentration of processing machineries and tools making difficult to find selling channels for crop diversification products
Power dynamics	Barriers referring to power dynamics characterizing the AES governance. This category relates to relations across actors within and outside the AES implementation process	4. Limited environmental advocacy representativeness at national level during policy negotiations
		5. Unbalanced representativeness of advocacy & lobbying groups in the decision-making process
Organizational	Barriers referring to challenges in coordination, planning, and network interactions among actors involved in AES implementation	6. Delays in AES subsidies disbursement to farmers
		7. Delayed provision to farmers of technical information on AES set of requirements
		8. Limited coordination across local and regional initiatives complementary to AES
		9. Lack of coherent environmental approach across local and regional initiatives
		10. Limited bottom-up feedback loops between policy implementation and policy design
		11. Limited coordination between administrative and budgetary responsibilities across public administration actors
Knowledge	Barriers referring to knowledge gaps impeding AES effectiveness. This category relates to knowledge gaps among various actor types	12. Limited knowledge on environmental issues among policy makers and elected members
		13. Limited knowledge of the effectiveness of AES, due to insufficient evaluation and science-based evidence
		14. Lack of multi-disciplinary skills and knowledge to advise on (feasible) farming practices to protect biodiversity
		15. Limited knowledge of available of public policy instruments & initiatives related to biodiversity protection
		16. Limited knowledge of consumers concerning biodiversity protection
		17. Limited networking across local and regional actors involved in biodiversity protection
		18. Limited knowledge of farmers on biodiversity and natural dynamics
		19.Limited knowledge of farming practices that are environmental and economically performing
Institutional	Barriers arising from structural, regulatory, and procedural constraints embedded within institutions	20. Lack of coherence of policy objectives versus long-term vision of local and regional development
		21. Restrictions to funding allocations (e.g., quota to advisory funding per farmer)
		22. Unstable and (often) changing political priorities
		23. Limited flexibility of national strategies and policies to heterogeneous territorial needs
		24. Lack of coherence between policy objectives and policy tools
		25. Potential exclusion of farmers due to the AES policy design
		26. Low compatibility of some AES with current farming practices
		27. Lack of environmental effectiveness of AES due to spatial mismatch
Financial	Barriers referring to financial aspects impeding AES effectiveness. This category relates to both financial constraints and financial allocation within the AES implementation process	28. Limited (and unstable) financial resources for AES implementation
		29. Limited (and unstable) financial resources for advising and supporting activities
		30. Limited added-value of sustainable farming practices within the value chain and final market prices
		31. Limited impact of market tools (e.g., price premium, standards, labels)
Cultural	Barriers referring to cultural aspects impeding adoption of AES, and their environmental effectiveness. This category relates to values, beliefs, and relational aspects	32. Social pressure from (part of) neighboring community discouraging AES adoption
		33. Limited communication between private and public actors of local and regional initiatives
		34. Lack of bottom-up approach in policy design (e.g., farmers inclusion in policy decision-making)
		35. Lack of trust by banks and other investments actors in environmentally friendly farming practices
		36. High responsibilities given to farmers to maintain public goods (e.g., healthy ecosystems)
		37. Productivity mindset as dominant mindset for farmers (and other actors of the agri-food system)
		38. Reticence of farmers to additional risks linked to AES
		39. Discouragement to adoption due to penalties in case of failure/irregularity of practice implementation
		40. Conflicting timescale of action between policy level and farming level

During the interviews, participants found it challenging to directly connect AES governance barriers to environmental performance, instead emphasizing broader scheme effectiveness and adoption issues. The list of barriers reflects this bias yet still offers valuable insights into factors influencing uptake and operational performance, shaping environmental outcomes, as AES is inherently designed to deliver environmental outcomes.

The list of barriers compiled throughout the interviews extended beyond the institutional aspects of AES governance, identifying challenges associated with roles and actors not previously considered within the AES governance process. This analysis revealed four additional roles and seven additional actors that, while not directly involved in translating policy frameworks into on-farm actions, nonetheless, according to the participants, influence AES’s environmental performance (Table 4). Such barriers touch upon a variety of issues from power dynamics of influence groups on policymaking, market value-added of AES, and consumer knowledge of AES measures or banks’ investment risk assessment practices.

**Table 4:** Additional roles and actor types linked to the barriers impeding AES in achieving environmental objectives

New role	New actor types
1. Advocacy	1. Value chain actors
2. Value chain integration	2. Consumers
3. Lobbying	3. NGOs
4. Investment	4. Farmers’ unions
	5. Research
	6. Education
	7. Investors

#### Linking barriers to the AES governance

**Fig. 5:**
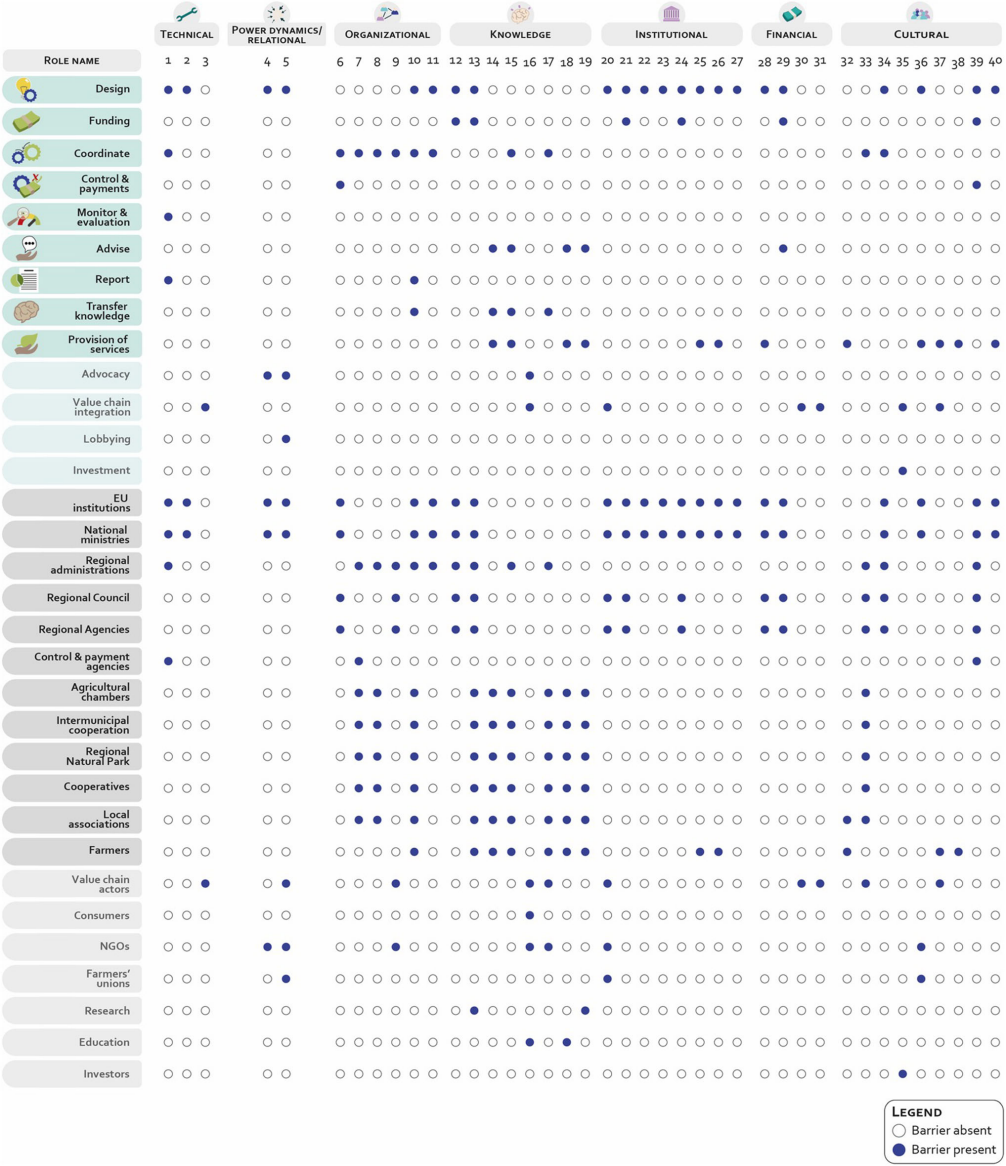
Boolean matrix providing information on the link between barriers and (i) AES roles and (ii) AES actors in Hauts-de-France; blue dots for presence of the barrier, white dots for absence of the barrier

Barriers within the AES governance process are unevenly distributed across both roles and actors (Fig. 5).

Knowledge barriers vary considerably depending on the specific knowledge gaps they create. These include a lack of technical knowledge of the biophysical and ecological processes underpinning AES measures, insufficient understanding of administrative and institutional procedures, and gaps in network knowledge, referring to an awareness of the actor networks involved in AES governance. Cultural barriers are similarly widespread, primarily affecting trust and social norms. During the interviews, some participants highlighted the challenges of maintaining a balance between ensuring accountability in public budget spending through control measures and fostering trust, which is crucial in voluntary programs such as AES.

Power dynamics and institutional, financial, and organizational barriers are less widespread across roles and actors. Power dynamics barriers relate to governmental actors involved in AES design and funding with advocacy and lobbying groups, highlighting the interplay between formal institutional governance and external influence groups. Additionally, during the interviews, some participants pointed out that AECP calls might suffer from some power dynamics. Underrepresentation of some associations as AECP leaders could occur, factoring in some political agenda instead of others.

Institutional barriers primarily affect actors engaged in EU, national, and regional policymaking. These barriers vary in nature and implications, ranging from design and funding constraints to broader agenda-setting challenges, such as ensuring coherence between AES objectives and the wider CAP policy framework.

Financial barriers are closely related to budgetary constraints and primarily affect funders and beneficiaries. They are also linked to value chain dynamics, as current market conditions often fail to capture and valorize farm-level efforts undertaken through AES measures in final products.

During interviews, participants also reflected on the flexibility of AES governance. Some expressed concerns that predefined national listings of measures might limit the relevance of measures at the regional level, as AES requirements are often fixed. Others, however, recognized a positive shift in the latest programming period, highlighting increased flexibility in AES measures to better align with regional needs and challenges.

Organizational barriers, in contrast, are more operational and predominantly affect administrative actors, particularly regional administrations. They involve challenges related to fund disbursement, coordination between administrations and local partners, and the lack of feedback mechanisms among actors engaged in AES governance.

**Fig. 6:**
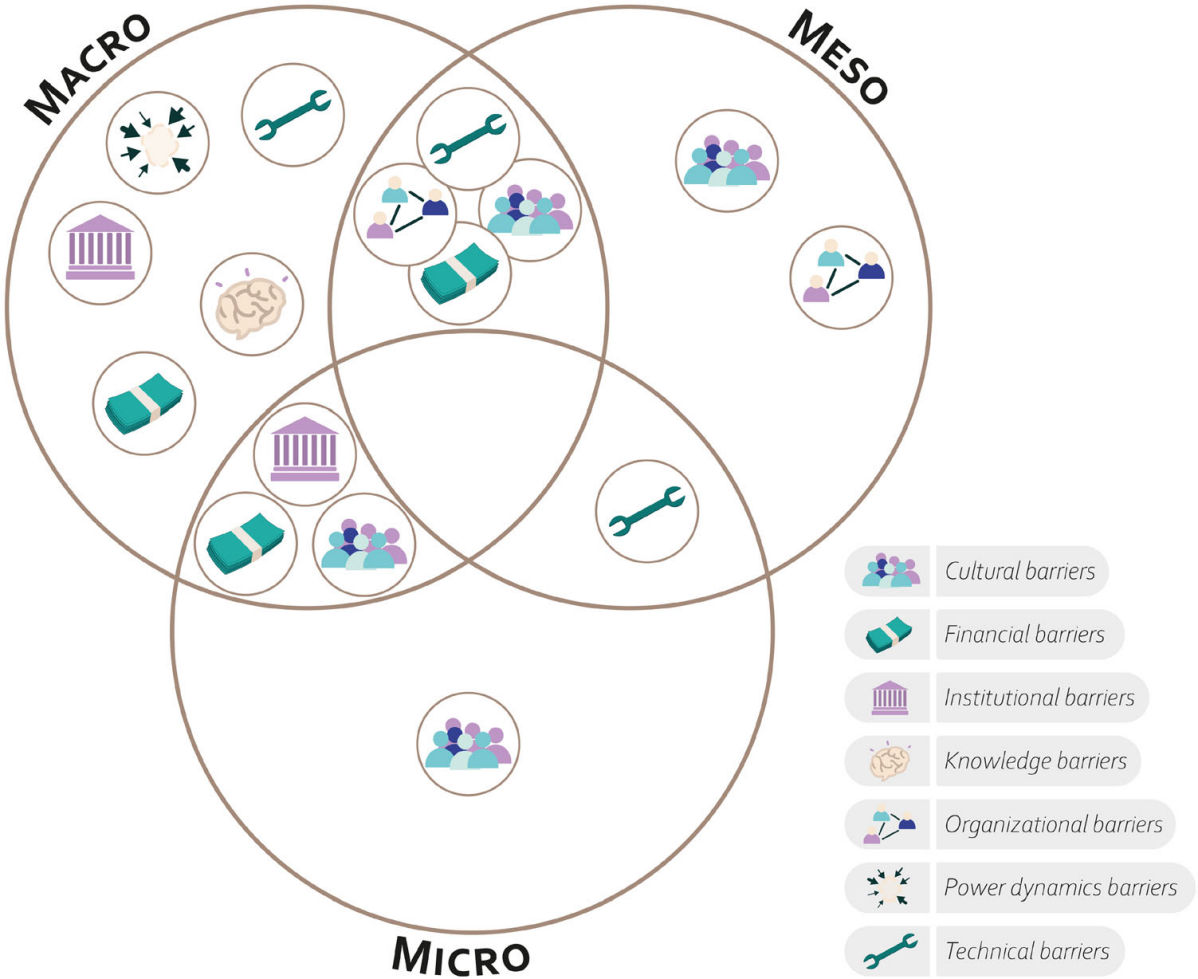
Barriers from a multilevel governance perspective, categories of barriers present per governance level in Hauts-de-France

From a multilevel governance perspective, barriers are closely intertwined with the multi-tiered nature of governance. Some barriers are specific to a single level, while others cut across multiple levels, underlying interdependency across levels (Fig. 6). A more detailed analysis of the barriers within each category reveals distinct patterns of constraints.

Barriers between the macro and mesolevels primarily derive from coordination issues. These include the limited involvement of mesolevel actors (especially at the macro–meso interface) in decision-making and the administrative burden imposed on public authorities regarding fund accountability. At the meso (especially at the meso–micro interface) and microlevels, shared barriers largely relate to knowledge gaps, both in ecological and biophysical processes and in the skills required to implement AES in a way that balances environmental and economic objectives.

The microlevel and macrolevel share institutional barriers linked to AES design, such as restrictive eligibility criteria that may exclude certain farmers. Cultural barriers also emerged, regarding the dominant policy narrative that places primary responsibility for environmental issues on farmers, shaping both perceptions and policy approaches.

Some barriers remain within a single governance level, a pattern observed at all three levels. The macrolevel faces a broad spectrum of barriers, ranging from restrictive AES requirements and power asymmetries driven by lobbying and advocacy networks to policymakers’ knowledge gaps on environmental issues and constraints in funding allocations. The mesolevel (especially at the macro–meso interface) is more affected by organizational barriers, such as delays in fund disbursement and coordination difficulties among regional initiatives, with the latter also reflecting cultural barriers that hinder communication among regional actors. At the microlevel, barriers are primarily cultural, including farmers’ risk aversion and the influence of local communities on individual decision-making.

From an actor-centered perspective, barriers do not always emerge within the roles where the mandated actors have the agency to address them. For instance, financial barriers related to AES budget allocation appear across funding, advisory, and service provision roles. However, decision-making authority over these financial constraints remains concentrated at the macrolevel, particularly among EU institutions, national ministries, and regional agencies responsible for fund distribution.

## Discussion

The analytical framework developed in this study helped move beyond simply listing factors that link governance and AES environmental performance. Instead, it allowed us to explore the interactions between actors, institutions, and processes within AES governance.

Our findings align with existing research. As Bazzan et al. (2023a,b) noted, knowledge exchange is a key issue at all governance levels. Our study adds to this by identifying different types of knowledge gaps depending on where they occur in the governance process. These range from knowledge gaps in administrative and institutional procedures occurring at mesolevel between public authorities and AES implementing actors to gaps in understanding biophysical and ecological processes at microlevel across both advisors and farmers, highlighting the need for targeted knowledge-sharing strategies. Institutional design issues in AES governance were another key point, consistent with critiques from Mettepenningen et al. (2013); Schomers et al. (2015); Toderi et al. (2017); de Vries et al. (2019). Problems with spatial targeting and contract structures can exclude certain farmers or lead to ineffective environmental measures. Power dynamics, as discussed by Beckmann et al. (2009) and Benoit and Patsias (2017), also emerged as a theme. However, unlike previous research which often focused on the unbalanced decision-making power between agricultural and environmental actors, our study highlights the influence of interest groups such as lobbying and advocacy networks. These groups often have uneven representation in policy negotiations, raising concerns about fairness and balance.

Our results also confirm coordination and stakeholder engagement challenges in AES governance (Toderi et al. 2017; Bazzan et al. 2023a,b). We show that these challenges differ across governance levels and require different strategies. At the mesolevel, tackling coordination and stakeholders’ challenges could involve building networks between regional agencies and local associations. At the macrolevel, addressing these challenges could involve feedback mechanisms between policymakers and administrations, reinforcing the macro–mesolevel interface.

A key takeaway from this study is that governance barriers are very diverse. Barriers of contrasted nature are found within each category (e.g., organizational, knowledge-based, and financial). Additionally, these barriers arise at different stages of AES governance process. Addressing them thus requires tailored strategies. For instance, solving a knowledge gap related to ecological processes differs from addressing gaps in administrative skills, even though both relate to knowledge. Additionally, some barriers remain within a single governance level while others span multiple levels. The latter entails that different actors and roles are involved, meaning that barriers at these intersections often require solutions that consider multiple perspectives. Recognizing this complexity is crucial when designing effective policy responses to those barriers.

Another important insight is that barriers affecting one group of actors cannot always be resolved at that level alone. For example, design and funding challenges affecting farmers’ adoption of AES require decisions at higher governance levels involving funding actors. If the barrier falls within the regional administration’s control, such as administrative burden in disbursement mechanisms, administrative simplification measures can be effective. However, administrative simplification alone is insufficient for budget allocations, which lie beyond the authority of regional administration. In such cases, negotiation and collaboration across governance levels are needed.

Finally, actors outside the governance structure also influence AES environmental performance. For example, market forces and consumer behaviors affect production decisions, meaning improving AES effectiveness requires looking beyond governance. Production and consumption dynamics need to be considered together.

The analytical framework developed in this study clarifies the relationships between governance levels, actors, and roles and links barriers with governance process. However, our interview protocol had limitations in assessing the link between barriers and environmental mechanisms, focusing more broadly on performance issues. We encourage future scholars to tackle this issue by, e.g., designing the interview protocol, integrating a set of questions, further challenging the set of barriers and their link to environmental performance, and investigating the underlying mechanisms.

## Conclusions

This paper developed an analytical and theoretical framework for systematically identifying and addressing governance factors hampering the AES environmental performance, validating it through a regional case study. Our findings explored the multilevel nature of AES governance and shed light on the complex web of barriers hampering the AES environmental performance. We identified six categories of barriers (i.e., technical, power dynamics & relational, knowledge, organizational, financial, cultural, and institutional) and emphasized their intertwined nature across the AES governance process. We found that some barriers are specific to a single governance level while others cut across multiple levels.

While some findings are region-specific, the study’s framework offers broader insights applicable to AES governance across EU Member States. First, addressing AES governance barriers requires accounting for their specific characteristics, which depend on the stage of the governance process in which they emerge. Second, actors facing the consequences of governance barriers do not always have full agency to resolve them. Opting for a lever without considering the nature of the barrier and the agency of the implementing actor could lead to ineffective strategies. Third, actors outside the governance process influence AES performance, and coordinating these external influences is essential to enhance the effectiveness of AES.

## Supplementary Information

Below is the link to the electronic supplementary material.

Supplementary file1 (XLSX 50 kb)

Supplementary file2 (PDF 376 kb)

## Data Availability

The data that support the findings of this study are included in the supplementary information (namely all data analysis steps, along with the data collection protocol and part of the data input). Some restrictions apply to the availability of the semi-directed interviews transcripts due to participant privacy agreements.
