# Supplementary material for: A governance perspective on agri-environmental schemes: Actors, roles, and barriers
Source: Ambio. 2025 Apr 19;54(11):1867–84. doi: 10.1007/s13280-025-02182-0 (PMC12480316; doi:10.1007/s13280-025-02182-0)
Supplement: Supplementary file 2 — Supplementary file2 (PDF 376 kb) [file 13280_2025_2182_MOESM2_ESM.pdf]

Ambio

Supplementary information

This supplementary information has not been peer reviewed.

Title: Agri-environmental schemes performance: the effect of the multi-level governance

Appendix S1. Additional information on data collection phase

The materials presented in this annex provides additional insights on the data collection phase the research team conducted in the implementation of both theoretical and analytical frameworks at the regional case study in Hauts-de-France. Namely, Table A1 displays the list of publication consulted during the initial phase of desktop research. Table A2 lists complementary on the expert consultation & semi-directive interviews conducted during the period March 2023 – August 2023, such as actor type per each participant and the date of the interview. Table A3 is the semi-directive interview protocol developed by the research team

Table S1.1

List of publications consulted during the desktop research phase

Authors	Date	Title	Topic	Type of publication
Martin et al.	2020	Diagnostic du système alimentaire des Hauts-de-France, de sa durabilité et de la résilience	Territorial analysis	Scientific publication
Juge, et al.	2019	Organic Flour in France	Organic production	Scientific publication
Guimont et al.	2018	Gérer l'urgence de la disparition du vivant : les contradictions temporelles de l'action publique	Public policy	Scientific publication
Matzdorf et al.	2010	How cost-effective are result-oriented agri-environmental measures? An empirical analysis in Germany	Agri-Environmental Schemes (AES)	Scientific publication
Uther & Matzdorf	2013	Studies on Agri-environmental Measures: A Survey of the Literature	Agri-Environmental Schemes (AES)	Scientific publication
European Commission	2022	Fiche d'information sur le programme de développement rural 2014- 2020 de Picardie (France)	Rural Development	Policy report
European Commission	2022	Fiche d'information sur le programme de développement rural 2014- 2022 du Nord-Pas-de-Calais (France)	Rural Development	Policy report
DRAAF	2022	Cartographie regionale des enjeux maec 2023-2027	Agri-Environmental Schemes (AES)	Policy report
DRAAF	2022	Cadrage regional maec 2023-2027	Agri-Environmental Schemes (AES)	Policy report
DREAL	2019	État des lieux dela biodiversité	Biodiversity	Policy report
DRAAF	2022	Memento 2022	Agricultural production	Policy report
Ministère de l'Agriculture et de l'Alimentation	2022	Plan Stratégique National de la PAC 2023-2027	Common Agricultural Policy	Policy document
Région Hauts-de-France	2020	Le Plan agro-écologie des Hauts-de-France 2020-2025	Agroecology	Policy document
Ministère de l'Agriculture et de l'Alimentation	2014	Guide méthodologique pour la mobilisation des mesures du FEADER en faveur du projet agro-écologique	Agri-Environmental Schemes (AES)	Methodological guidelines
Ministère de l'Agriculture et de l'Alimentation	N/A	Call AECP	Agri-Environmental Schemes (AES)	Policy document

Table S1.2

List of participants for both expert consultation and interviews

ID	Actor type	Type of interaction	Date
1	Research institute	Expert consultation	29/03/2023
2	Think tank	Expert consultation	30/03/2023
3	Think tank	Expert consultation	30/03/2023
1	Local association	Interview	30/03/2023
2	Regional administration	Interview	21/04/2023
3	Regional Natural Park	Interview	16/05/2023
4	Regional agency	Interview	17/05/2023
5	NGO/Local association	Interview	17/05/2023
6	Agricultural Chamber	Interview	22/05/2023
7	Regional administration	Interview	02/06/2023
8	Regional administration	Interview	13/06/2023
9	Regional Council	Interview	14/06/2023
10	Control & payment Agency	Interview	19/06/2023
11	Regional Agencies	Interview	23/06/2023
12	Regional administration	Interview	04/07/2023
13	Cooperative	Interview	11/07/2023
14	Local association	Interview	25/07/2023
15	Agricultural Chamber	Interview	25/07/2023
16	Local association	Interview	17/08/2023
17	Cooperative	Interview	30/08/2023

Table S1.3

Semi-directive interview protocol

Section	Minutes	Question	Output
1. Introduction	10’	Can you provide a brief introduction of yourself and your organization?	Description of the interviewee<br>Description of the organization
2. AES governance	45’	Can you provide a description of the role of your organization in AES implementation process?	Description of the role of the organization
		With which actors do you exchange?	AES governance process
		With whom you collaborate?	
		Which type of relationship you have with them (i.e. knowledge exchange, financial exchange)?	
3. Identification of the AES barriers	35’	What are the barriers you could think of regarding the environmental performance of AES?	List of barriers
		How would you explain the mechanisms leading to these barriers?	Mechanisms behind barriers

Appendix S2. Background research and additional material on AES governance

AES governance has been already targeted by recent studies, and our study is grounded in this literature. The classification of AES roles has been extensively documented by the recent publication (Sattler et al., 2023)[1], and we use this as the key grounding reference of our theoretical and analytical frameworks. Some of the role's description has been adapted or merged. Their listing of roles is presented in table B1. As the second main component of the AES governance process are the actors, in table B2 we provide a description of each actor type that has been identified during the interviews as playing a role in the overall AES governance process. Table B3 show the table developed by the research team summarizing the main insights collecting during the interview phase and validated during the seminar, concerning the links between actors and roles.

Table S2.1

Full list of roles

ROLE NAME	ROLE DESCRIPTION AS PROVIDED BY SATTLER[1] ET AL., 2023	ADAPTED DESCRIPTION
Design	Actors who were/still are involved in the negotiation and the design of the contract, including the definition of environmental goals (targeted ES) and the possible measures to achieve them	N/A
Funding	Actors who provide (public or private) funding for the contract	N/A
Coordination	Actors who coordinate the efforts of the other actors involved in the provision of the targeted ES	Actors responsible for translating AES policy design and their implementation , and coordinating the efforts of involved actors
Monitoring	Actor who perform monitoring tasks at site, field or landscape scale	N/A
Evaluation	Actors who are involved in the evaluation of the contract, where the outcomes of this evaluation have consequences for the (dis)continuation and the further design of the contract	N/A
Payment and administration	Actors who handle the administration of the payments	**Control & payments**: Actors responsible for conducting random or scheduled controls at the site, field, or landscape scale to verify compliance with agreed measures, overseeing sanctions when necessary, and handling the administration of payments
Controlling/ sanctioning	Actors who conduct random/scheduled controls at site, field or landscape scale to verify that the contract actors perform all agreed measures, and are involved in the sanctioning of respective parties	
Reporting	Actors who are involved in the documentation and reporting of the environmental and other results/outcomes of the contract	N/A
Advice/extension	Actors who give advice/provide extension services to the contracted actor and/or other actors involved in the contract	Actors who provide advisory support to farmers for the implementation of AES at the farm level
Knowledge	Actors who are involved in knowledge provision, pooling, exchange and distribution	**Transfer knowledge**: Facilitating the exchange of ecological and biophysical knowledge for implementing AES and ensuring an effective information flow for reporting between governance levels and farmers.
ES seller	Actors who are contracted (by EU buyers) to provide and sell ecosystem services (ES) targeted by the contract, typically farmers or other land managers.	**Provision of services**: Actors that ensure the management decisions that align with scheme requirements, compliance with contractual obligations and eventually environmental outcomes

[1] Sattler, C., Barghusen, R., Bredemeier, B., Dutilly, C., & Prager, K. (2023). Institutional analysis of actors involved in the governance of innovative contracts for agri-environmental and climate schemes. *Global Environmental Change*, *80*, 102668. https://doi.org/10.1016/j.gloenvcha.2023.102668

Table S2.2

Matrix linking actor types and roles in the regional case study in Hauts-de-France (FR)

Actors' type	Regional scale	Design	Funding	Coordinate	Control & payments	Monitor & evaluation	Advise	Report	Transfer knowledge	Provide services	**Total roles per actor**
EU Institutions	EU	1	1	0	0	0	0	0	0	0	2
National Ministries	National	1	1	0	0	0	0	0	0	0	2
Regional Administrations	Regional	0	0	1	0	1	0	0	0	0	2
Regional Council	Regional	0	1	1	0	0	0	0	0	0	2
Regional Agencies	Regional	0	1	1	0	0	0	0	0	0	2
Control & payment Agencies	Regional	0	0	0	1	0	0	0	0	0	1
Agricultural Chambers	Regional & Departmental	0	0	0	0	0	1	1	1	0	3
Public intercommunal cooperation establishment	Departmental	0	0	0	0	0	1	1	1	0	3
Regional Natural Park	Regional	0	0	0	0	0	1	1	1	0	3
Cooperatives	Departmental	0	0	0	0	0	1	1	1	0	3
Local associations	Departmental	0	0	0	0	0	1	1	1	0	3
Farmers	Farm	0	0	0	0	0	0	0	1	1	2
**Total actors per role**		2	4	3	1	1	5	5	6	1	

Table S2.3

List of actors

Actor type	Description
1. EU Institutions	The European Union (EU) is governed by several institutions that collectively shape and implement public policy across its member states and across various policy areas. These include the European Commission, which proposes and enforces legislation; the European Parliament and the Council of the European Union, which jointly pass laws and approve budgets; and the European Council, which sets the EU's overall political direction.
2. National Ministries	Governmental bodies responsible for the formulation, implementation, and oversight of public policies across various sectors such as food and agriculture, or the environment. Each ministry, led by a Minister, develops national strategies, allocates resources, and coordinates with regional and local authorities to ensure that policies are effectively implemented throughout the country.
3. Regional Administrations	Regional delegates of the national ministries, and each ministry has its regional delegated staff. It is responsible of the implementation of national policies. It works often in collaboration regional delegates of other ministries, and other regional and local actors
4. Regional Council	The elected body responsible for governing and managing various regional matters. Even if agriculture is mainly under the decision making of national policy, the regional council allocates some fundings to the achievement of objectives under the interest of the regional agricultural dynamics. It often collaborates with the regional delegates of the ministries and other actors at local level.
5. Regional Agencies	Decentralized public bodies that operate at the regional level to implement national policies tailored to local needs. They are responsible for managing various aspects of public services and environmental resources within their regions, including water management, public health, and environmental protection. These agencies work closely with local authorities, industries, and communities to ensure that national objectives are met while addressing specific regional challenges and priorities. They typically manage a combination of direct revenues—such as fees and charges related to their specific functions—and government subsidies, allowing them to operate with a mix of financial autonomy and state support.
6. Control & payment Agencies	The public service that is responsible for the validation of the subsidies accessible to farmers. Is the main actor implementing the administrative burden and represent on the direct connection between the administrative actors and the farmers. They deal with compliance mainly, but they are not the organisation responsible for the payments to the farmers.
7. Agricultural Chambers	Representative organization of the interest of farmers (but not only), it does farm advising activities and plays quite an active role in the community.
8. Intermunicipal cooperation	Administrative structures that enable several municipalities to exercise joint powers.<br>They are subject to common rules that are uniform and comparable to those of local authorities. EPCIs include urban communities, conurbation communities, communities of communes, new conurbation syndicates, communes' syndicates and mixed syndicates.
9. Regional Natural Parks	The public entity responsible for the management of the area under the park and also has some responsibilities concerning the actors interacting within the territorial boundaries of the park (e.g. activities of farm advising).

10. Cooperatives	Organizations where producers pool resources, purchase supplies, and market their agricultural products collectively. They are structured as cooperative societies with farmer-members who own shares and participate in decision-making. Their role in agri-environmental schemes and related measures includes advising farmers on sustainable practices and intermediaries with public administrations
11. Local associations	Organizations are often structured as non-profit entities focused on environmental protection and community engagement, that are as well intermediaries between farmers and public administration facilitating farmer participation to AES measures, and sometimes offering technical advice
12. Farmers	Individual engaged in the cultivation of crops or the raising of livestock for the purpose of producing food, fiber, or other agricultural products
